# Effects of temporary sacral nerve stimulation on gastrointestinal motility and function in patients with chronic refractory slow-transit constipation

**DOI:** 10.1007/s10151-020-02367-7

**Published:** 2020-11-13

**Authors:** D. F. Altomare, A. Picciariello, A. Di Ciaula, M. Rinaldi, M. De Fazio, P. Portincasa

**Affiliations:** 1 Department of Emergency and Organ Transplantation, University “Aldo Moro”, Bari, Italy; 2 Department of Biomedical Sciences and Human Oncology, Clinica Medica “A. Murri”, University “Aldo Moro”, Bari, Italy; 3 IRCCS Istituto Tumori Giovanni Paolo II, Bari, Italy

**Keywords:** Chronic slow-transit constipation, Sacral nerve stimulation, Gastrointestinal motility, Autonomic nerve function

## Abstract

**Background:**

The efficacy of sacral nerve stimulation (SNS) on patients with chronic refractory slow-transit constipation is controversial and its mechanism of action on gastrointestinal motility and transit is not fully understood. The aim of this study was to document the effects of temporary SNS on the gastrointestinal and biliary tract motility and on gastrointestinal transit in patients with refractory slow-transit constipation.

**Methods:**

This was a prospective interventional study. Patients with slow-transit chronic constipation, unresponsive to any conservative treatment, were enrolled between January 2013 and December 2018. Patients’ quality of life [patient assessment of constipation quality of life (PAC-QOL) questionnaire], constipation scores (Cleveland Clinic Constipation Score) colonic transit time (CTT), orocecal transit time (OCTT), gastric and gallbladder kinetics, together with the assessment of the autonomic nerve function were evaluated before and during temporary SNS.

**Results:**

14 patients (12 females, median age 38 years, range 24–42 years) had temporary SNS. The Cleveland Clinic Constipation Score did not change compared to baseline (23 ± 3 vs 21.4; *p* = 070). The PAC-QOL did not improve significantly during the stimulation period. Gallbladder/stomach motility (half-emptying time) did not change significantly before and after SNS. OCTT was delayed at baseline, as compared to standard internal normal values, and did not change during SNS. CTT did not improve significantly, although in two patients it decreased substantially from 97 to 53 h, and from 100 to 65 h.

**Conclusions:**

Temporary SNS did not have any effect on upper/lower gastrointestinal motility and transit in patients with severe constipation.

## Introduction

Sacral nerve stimulation (SNS) is a minimally invasive, reversible and low-risk procedure currently considered an effective and reliable treatment option for patients with urinary and fecal incontinence of various etiologies [1], with positive long-term outcomes [2].

SNS also improves other pelvic floor dysfunctions such as urinary retention [3], and constipation [4]. In particular, studies suggest improvement in constipation symptoms after SNS in both slow-transit and obstructed defecation, with success rates which vary from 22 to 73% depending on the length of the follow-up, irrespectively of the type of constipation [4–11]. However, the mechanisms of action of SNS have not yet been entirely elucidated.

The peripheral effects of SNS on the target organs do not fully explain the functional outcome of the procedure. One possibility is the involvement of higher neurological centers (spinal and brain) [12, 13]. The autonomic nervous system might also play a role in the beneficial effects observed following SNS in patients with functional bowel disorders [14]. In fact, slow-transit constipation is often the most relevant clinical expression of a pan-enteric motility disorder involving the whole gastrointestinal (GI) tract and the biliary motor function [15, 16], with potential impact on the outcome of both medical and surgical treatments [17]. At the moment, the effects of SNS on motility and transit in patients with severe constipation is poorly documented. Therefore, there is no clear evidence supporting the role of this minimally invasive, but expensive treatment in these patients.

The aim of this prospective study was to document the effects of temporary SNS on GI and biliary tract motility and on GI transit in patients with refractory slow-transit constipation. We also evaluated the potential role of an underlying subclinical autonomic neuropathy (AN) in the response of constipated patients to SNS.

## Materials and methods

### Patients

The study was approved by the local ethics committee of the Azienda Ospedaliero-Universitaria Policlinico of Bari, Italy and all the patients gave their written informed consent to enter the study.

Patients attending our tertiary referral center of colorectal surgery between January 2013 and December 2018, for chronic constipation unresponsive to any conservative treatment were offered a SNS test as an off-label treatment before considering any surgical option.

The inclusion criteria were

- presence of chronic refractory slow-transit or mixed constipation according to the Rome III criteria [18] and lasting more than 12 months;
- patients unresponsive to any conservative treatment (including adequate dietary regimen, oral laxatives/prokinetics, even in high doses, enemas), and showing a significant deterioration of their quality of life (QOL) (≤ 50% of normal value of the patient assessment of constipation quality of life [PAC-QOL] questionnaire [19]);
- patients with a documented slow-transit constipation (colonic transit time with the radiopaque marker methodology > 90 h [20]) and a negative colonoscopy/barium enema performed within the last year.

The exclusion criteria were

- obstructed defecation due to rectocele and/or rectal intussusception, paradoxical puborectalis syndrome as documented by dynamic proctography;
- any neurological diseases involving the central nervous system, inability to collaborate and understand the procedure;
- irritable bowel syndrome (as confirmed by the Rome III criteria [18]);
- inflammatory bowel disease;
- other conditions including pregnancy, severe liver or renal diseases, congenital coagulative defects, hypoganglionosis or Hirschsprung’s disease;
- use of drugs affecting gastrointestinal motility, including anti-Parkinson drugs;
- sacral abnormalities preventing safe and effective positioning of the sacral electrode.

### Study design

This was a prospective study. At baseline, we recorded the full clinical history, performed the proctological evaluation, and assessed autonomic nerve function using the heart rate variability test and the sweat spot test (see below). Before and during temporary SNS, we evaluated patients’ quality of life (PAC-QOL questionnaire), constipation scores (Cleveland Clinic Constipation Score), gastrointestinal motility, gallbladder and gastric emptying (functional ultrasonography), orocecal transit time (H_2_-lactulose breath test, OCTT), and colonic transit time ([CTT]radiopaque markers).

### Nerve evaluation test

The temporary nerve evaluation test was performed under local anesthesia by implanting a quadrupole tined lead electrode (tined lead; Interstim 3889–28 cm; Medtronic^®^ Inc. Minneapolis, MN, USA) into the S3 foramen connected with an extracorporeal electrostimulation for 4 weeks. The correct placement of the electrode in the third sacral foramen was confirmed by X-ray and by testing the patient’s perception of the electrical stimulation in the anal-perineal region. The voltage for the electrostimulation was set at just above the subjective sensation of the perineal/anal vibration with a standard frequency of 19 Hz. The treatment was considered successful if the number of bowel movements/week and the patient’s subjective evaluation of symptoms improved > 50%, compared to baseline. In successful cases, an internal permanent pulse generator (IPG) (Interstim 3057-6SC; Medtronic^®^ Inc., Minneapolis, MN, USA) was implanted under local anaesthesia.

### Clinical evaluation

The clinical assessment included a bowel diary and the Bristol stool scale [22] to evaluate bowel function, the Cleveland Clinic Constipation Score (CCCS) [21] for the severity of symptoms and the PAC-QoL questionnaire [19].

The severity of constipation was scored by the 30-point CCCS questionnaire which includes eight items scored 0–4 (excluding the item “assisted defecation” scored 0–2) according to their severity. Quality of life was assessed by the PAC-QOL questionnaire [19]. This patient-centered and self-administered questionnaire includes 4 domains (worries and concerns, physical discomfort, psychosocial discomfort, and satisfaction) with a total of 28 items scored 0–4 according to their severity. To document the potential effects of SNS, the assessments of the CCCS, PAC-QOL, OCTT were carried out after at least 2 weeks of temporary SNS.

### Assessment of the autonomic nerve function

The presence of an AN was determined by two validated tests.

**Fig. 1:**
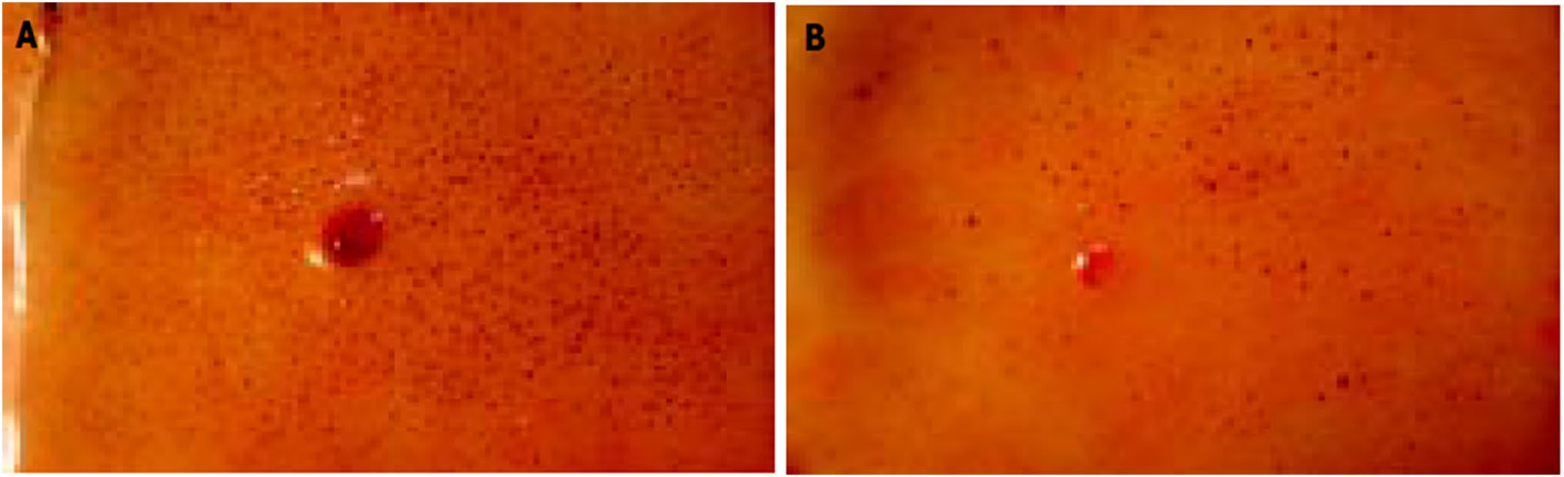
Normal (**a** even distribution of numerous workings sweat glands) and abnormal (**b** rare and uneven distributed sweat glands) sweat spot test after thermal stimulus

The sweat spot test [23] explores the involvement of cholinergic sympathetic fibers. Briefly, an iodine and a fine emulsion of starch in arachis oil is applied to the dorsal skin of the foot. When the sweat is secreted from the skin glands under a thermal stimulus, the water contained in the sweat drops triggers a chemical reaction between the iodine and the starch making each sweat gland pore visible as a brown dot (Fig. 1). To evaluate the test, the number and distribution of dots appearing in a standard squared grid of 529 mm^2^ divided into 64 squared subareas is measured. If there are at least 12 dots/subarea and/or < 8% of abnormal subareas (each square of the grid having less than 6 dots), the test result is normal. The heart rate variability (HRV) test [24] evaluates the involvement of parasympathetic fibers, and is performed using time-domain methods**.** During an electrocardiogram (ECG) in a quiet room, normal beat-to-beat normal-to-normal (NN) or RR intervals, i.e., intervals between adjacent ORS complexes, are analyzed. Variables include: standard deviation of NN intervals (SDNN) (over a 24-h period), standard deviation of the average NN intervals calculated over short periods (5 min) (SDANN), square root of the mean squared difference of successive NNs (RMSSD), the number of pairs of successive NNs that differ by more than 50 ms (NN50), and proportion of NN50 divided by total number of NNs (pNN50). SDANN becomes a measure of changes in heart rate due to cycles longer than 5 min. Thus, SDNN reflects all the cyclic components responsible for variability in the period of recording; therefore, it represents total variability. The HRV was, therefore, expressed by the low-frequency (LF) activity/high-frequency (HF) activity ratio (normal values < 2.0).

### Test meal

The standard test meal (Nutridrink^®^; Nutricia, Milan, Italy) consisted of 200 mL liquid suspension containing 12 g (20%) protein, 11.6 g (19%) fat, and 36.8 g (61%) carbohydrates for a total of 300 kcal, 1260 kJ, 455 mOsm/L. Lactulose (10 g = 15 mL Lattulac^®^, SOFAR, Trezzano Rosa, Milan, Italy) is added to the test meal for allowing cecal fermentation for the estimation of orocecal transit time (OCTT). The final volume of the meal was, therefore, 215 mL. The test meal volume and the amount of fat/lactulose are sufficient to induce a valid gastric and gallbladder response by 120 min, and small intestinal response by 180 min [25].

### Gastric and gallbladder kinetics

Coordinated gastric dilation/emptying and gallbladder emptying/refilling were studied simultaneously by functional ultrasonography, as described elsewhere [25]. The fasting subject in a sitting position following the ingestion of the standard liquid meal, consumed within 2 min. Time-dependent changes of fasting and postprandial gallbladder volumes (mL) and antral areas (cm^2^) are subsequently calculated from frozen sonograms on a portable scanner (Noblus, Hitachi Medical, Tokyo, Japan) equipped with a 3.5 MHz convex transducer. Gallbladder volume and antral area are measured before the meal at − 10, − 5 and 0 min and after the meal every 5 min during the first 30 min and every 15 min thereafter up to 120 min. Indices of gallbladder and gastric kinetics were summarized as half-emptying time (T1/2, min) calculated by linear regression analysis from the linear part of the emptying curves. T1/2 was the time at which 50% decrease of gallbladder volume and antral area were observed. Additional parameters were gallbladder fasting and residual volumes (in mL) and gastric basal antral area and maximal antral area (as cm^2^).

### OCTT

OCTT was measured by standard guidelines [26].

During the 10 days before the test, antibiotics, probiotics, or other drugs known to affect gastrointestinal motility or intestinal microbiota were prohibited. The patient’s diet the day before the test consisted of meat, fish, eggs and olive oil, and water to drink (no fermentable carbohydrates). Breath samples were taken before the meal and, subsequently, every 10 min up to 180 min after the ingestion of the meal, during which a rise of 10 p.p.m. above baseline on two consecutive measurements (i.e., OCTT in minutes) was observed in all patients. Time-dependent changes of H2 in expired breath were assessed using a pre-calibrated, portable hydrogen-sensitive electrochemical device (EC60-Gastrolyzer; Bedfont Scientific, Medford, NJ, USA). Results were expressed as H_2_ excretion in parts per million (p.p.m.), with a detector accuracy of ± 2 p.p.m.

### CTT

CTT was evaluated by the radiopaque markers test described by Metcalf et al. [20]. Briefly each patient ingested capsules containing 20 radiopaque markers and the same time for the first 3 days and make an abdominal X-ray on the 4th and 7th day at the same time. The number of markers retained is counted on the 2 X-rays and multiplied by the constant 1.2 to provide transit as hours (normal value < 74 h). The diet must remain unchanged, and no laxatives or enemas are allowed during the whole week of the test.

### Statistical analysis

Data were expressed as mean ± SD or median ± ranges and compared by the Student’s *t* test or the Wilcoxon’s rank sum test for paired data. Differences in the emptying curves were evaluated by two-way ANOVA repeated-measures, followed by Fisher’s LSD multiple comparison test. Medcalc statistical software version 19.1 was used for the statistical evaluation. A *p* value < 0.05 was considered statistically significant.

## Results

14 patients (12 females, median age 38 years, range 24–42 years, median body mass index = 23 kg/m^2^, range 21–27 kg/m^2^) entered the study. The median duration of constipation was 14 years (range 8–16 years). The mean number of bowel movements/week was 1.1 ± 0.6, with a mean Bristol Scale score of 1.5 ± 0 and a baseline CCCI value of 23 ± 3. All enrolled patients were unresponsive to dietary and behavioral changes, osmotic laxatives, sennosides, prucalopride and even to enemas or hydrocolontherapy. No complications were observed and all the patients were discharged the same day of the temporary SNS procedure.

All the enrolled patients completed all the steps of the temporary SNS. Three patients showing significant benefits from the electrostimulation underwent permanent SNS by an implantable pulse generator. Two of these patients have still some benefit from SNS while the third patient’s implant was removed because of early postoperative recurrence of symptoms.

Subclinical signs of AN were detected in all patients. The mean SST was 7.2 ± 10 mean SST score of (range 0.8–14.0). (normal value 12 dots/subarea) and the HRV (low-frequency activity/high-frequency activity) was 2.7 ± 3 (normal value < 2.0).

The number of bowel movements/week and the Bristol stool scale did not change significantly during the 4 weeks of temporary SNS compared to the baseline (1.6 ± 0.7 compared to 1.1 ± 0.6 bowel movements/week, *p* = 0.07) (Table 1). However, in three patients it improved, on average, from 1 to 2 bowel movements/week although the upper GI motility investigations did not differ from those of the rest of the patients. The severity of the CCCS did not change compared to the baseline (23 ± 3 vs 21.4; *p* = 070). Similarly, the QOL of these patients during the stimulation period, measured by the PAC-QOL did not improved significantly.

**Table 1:** Clinical and instrumental evaluation of temporary SNS in 14 patients with chronic, refractory slow-transit constipation (data expressed as mean ± SD)

	Baseline	SNS ON	*p*
Bowel movements/week	1.1 ± 0.6	1.6 ± 0.7	0.07
Bristol stool score	1.5 ± 0	1.6 ± 0.5	0.80
Cleveland Clinic Constipation Score	23 ± 3	21 ± 4	0.70
Gastric half-emptying time (T50, min)	48.8 ± 7.1	43.5 ± 7.4	0.23
Gallbladder half-emptying time (T50, min)	28.5 ± 4.2	35.2 ± 4.5	0.07
OCTT (min)	141.7 ± 102	144 ± 115	0.97
CTT (h)	95.3 ± 37	91.2 ± 50.7	0.09
PAC-QOL	2.96 ± 0.52	2.4 ± 0.83	0.09

*SNS* sacral nerve stimulation, *OCTT* orocecal transit time, *CTT* colonic transit time, *PAC-QOL* patient assessment of constipation quality of life

The gallbladder/stomach motility (half-emptying time) did not change significantly (28.5 ± 4.2 min vs 35.2 ± 4.5 min, *p* = 0.07; 48.8 ± 7.1 vs 43.5 ± 7.4 min, *p* = 0.23, respectively) before and after SNS. Other parameters of kinetics did not change either, including gallbladder fasting volume (23.5 ± 2.1 mL vs 22.4 ± 1.8 mL, respectively), residual volume (9.4 ± 1.1 mL vs. 10.1 ± 0.5 mL, respectively), and gastric antral area at baseline (4.2 ± 0.4 cm^2^ vs 4.7 ± 0.6 cm^2^, respectively) and maximal antral area (11.2 ± 0.8 cm^2^ vs 13.7 ± 0.7 cm^2^, respectively). OCTT was delayed at baseline, as compared to standard internal normal values (111 ± SEM min as assessed in a group of 24 healthy subjects) [25], but did not change during SNS (141.7 ± 102.6 vs 144 ± 115.2, *p* = 0.97). The CTT did not improve significantly, although in two patients it decreased substantially from 97 to 53 h, and from 100 to 65 h.

## Discussion

This is a comprehensive study dealing with patients suffering from severe chronic constipation refractory to conservative treatment. We explored GI motility, severity of symptoms, QOL, autonomic nervous system function in relation to the outcome of SNS, and to the best of our knowledge, this has not been studied before.

The treatment of severe refractory slow-transit constipation is a complex and challenging task for gastroenterologists and colorectal surgeons. The decision to perform destructive, irreversible and risky surgery such as total/subtotal colectomy is made with great reluctance by surgeons, because of the uncertainty of its functional outcome [27]. This type of constipation may be in fact the tip of the iceberg represented by a pan-enteric AN involving the whole GI tract [15]. Taking into account these considerations, any alternative treatment, such as SNS, could be justified, even if off-label.

SNS has become an established therapeutic option for fecal and urinary incontinence over the past decade [2, 3] and, although its mechanism of action is still poorly understood, the observation of some beneficial effects on patients with the opposite pelvic floor dysfunction, constipation and urinary retention, have justified its application to treat these diseases.

Several studies and even a metanalysis exist on SNS for chronic constipation. While initial reports, on the wings of the enthusiasm around SNS, show a high success rate (over 70%) [5], almost all the subsequent studies have reported disappointing results after medium-/long-term evaluation, with a success rate around 30% [6–11].

However, few papers investigated the pathophysiological mechanism underlying the possible effects of SNS on GI motility and transit in patients with severe constipation.

Our study explored upper GI motility and the GI transit in patients with slow-transit constipation before and during temporary SNS, by measuring the gallbladder and gastric emptying time and the OCTT. No changes from baseline were observed during temporary SNS.

Similar studies have been carried out on patients with fecal incontinence having ON/OFF SNS using scintigraphy or an experimental magnetic tracking system, and showed no changes, from baseline, in agreement with our findings [12, 13].

However, our results showed a normal gallbladder and gastric emptying time but a delayed OCTT, without any change during temporary SNS. A delayed OCTT could derive from a subclinical AN, which was demonstrated in all the patients selected in our study using the sweat spot test and the HRV test. In fact, a progressive deterioration of the sweat glands is one of the earliest detectable neurophysiologic abnormalities in distal small-fiber neuropathy causing pseudomotor dysfunction [15].

The effects of temporary SNS on colonic motility in constipated patients were investigated in only two papers from the same group of investigators. In the first study [28] on eight patients, an increase of propagated high amplitude contractions were documented during temporary SNS using 24-h pancolonic manometry, accompanied in some of them by symptom relief. In a second study [29] on nine patients, these effects on colonic motility were documented during a supra-sensory stimulation, but not during sham stimulation, or sub-sensory stimulation. However, no change in the number of bowel movements/week was documented. Due to the wide inter- and intra-variability of colonic mass movements over the time and to the very low number of patients recruited, no clear conclusions can be drawn from these studies.

The most relevant investigation in these patients should be the assessment of the colonic transit time, which could be related to the number of bowel movements/week and, possibly, to changes in the Bristol stool scale.

Before our study, the effects of SNS on colonic transit time in patients with chronic constipation have been evaluated in only four papers using radio-opaque markers. The first of these studies [5] was a multicentre, prospective trial on 27 constipated patients. Results showed an increased median number of bowel movements (from 2.7 to 6.5/week) in those who reported an improved CTT under SNS. However, several considerations cast doubts on the validity and generalizability of the reported data, including the inclusion criteria. In fact, patients who responded positively to SNS had a baseline median number of bowel movements/week of 2.7, which was very close to the normal range.

A more recent study did not show any improvement in the colonic transit time in 11/20 constipated patients after 1 year of SNS [30].

In line with these papers, in our study the median CTT did not change significantly during temporary SNS in our series of 14 patients, even if in 3 of them an improvement in bowel movement frequency was reported.

Another point of interest is the potential effects of SNS on subjective symptoms, and in particular on QOL, which is severely disturbed [31]. In our study, the QOL evaluated by a dedicated questionnaire for constipation and the severity of the disease evaluated by the CCCS did not change. This matches well with the unchanged GI motility and bowel movements/week. Despite these findings, three of our constipated patients reported a symptomatic improvement of the constipation, and were consequently implanted with a permanent SNS. The long-term outcome of these patients is now available, showing that two of them (both young females, candidates for total colectomy) are still using the SNS with moderate benefits. However, a placebo effect and the willingness to believe in an advanced therapy like SNS could explain these findings [33, 34]. The effects of depressive disorders demonstrated by the MMPI questionnaire in the response rate to SNS has also been considered recently, and it has been shown that a minority of constipated patients with normal MMPI could potentially benefit from permanent SNS [32].

A limitation of our study is the small sample size due to the low number of patients fulfilling our inclusion criteria and willing to undergo SNS. Nevertheless, all the patients recruited completed the study.

## Conclusions

Our study demonstrates that there is no proof of any effect of temporary SNS on upper/lower GI motility and transit in patients with severe constipation. The role of undetected AN must be taken into account in the selection of treatment for these patients This type of patient is likely a poor responder to any kind of therapy.
